# The effect of damp housing on psychological distress: does respiratory health matter?

**DOI:** 10.1093/aje/kwag042

**Published:** 2026-02-28

**Authors:** Maria Rosa Gatto, Ang Li, Erika Martino, Rebecca Bentley

**Affiliations:** Healthy Housing, Centre for Health Policy, Melbourne School of Population and Global Health, The University of Melbourne, Parkville, VIC 3010, Australia; Healthy Housing, Centre for Health Policy, Melbourne School of Population and Global Health, The University of Melbourne, Parkville, VIC 3010, Australia; Healthy Housing, Centre for Health Policy, Melbourne School of Population and Global Health, The University of Melbourne, Parkville, VIC 3010, Australia; Healthy Housing, Centre for Health Policy, Melbourne School of Population and Global Health, The University of Melbourne, Parkville, VIC 3010, Australia

**Keywords:** damp, housing, mental health, respiratory, effect modification

## Abstract

Damp housing is associated with poor mental health. However, it is unknown whether people with chronic respiratory conditions (CRCs) have increased risk of negative mental health effects, given their increased susceptibility to dampness-related physical health effects. Data from the British Household Panel Survey were used to quantify the differential effect of damp housing exposure on psychological distress by CRC status. Adjusted fixed effects logistic regression models stratified by CRC were performed, followed by models testing for statistical interaction. In stratified models, people living with a CRC at baseline reported greater odds of psychological distress associated with damp housing (OR = 1.27, 95% CI: [1.14, 1.41], *P* < .01) compared with people in good respiratory health (OR = 1.07, 95% CI: [1.02, 1.12], *P* = .01). There was weak evidence of effect modification by change in CRC status (interaction term OR = 1.09, 95% CI: [0.98, 1.20], *P* = .10). However, there was strong evidence of effect modification by baseline CRC status (interaction term OR = 1.19, 95% CI: [1.06, 1.34], *P* < .01). Our analysis suggests that remediating sources of dampness in the home may alleviate some of the mental toll of living with a CRC.

## Introduction

Globally, 545 million people have a chronic respiratory condition (CRC) such as asthma, chronic obstructive pulmonary disorder (COPD), and chronic bronchitis.^1^ Exposure to damp housing is a well-established trigger of respiratory symptoms, asthma onset, and exacerbation of existing respiratory conditions.^2^^-^^4^ There is a high prevalence of poor mental health among people with respiratory conditions.^5^^-^^7^ Evidence suggests that people with respiratory conditions have good knowledge of their triggers and make a range of lifestyle adjustments to cope.^8^ However, awareness of triggers can induce anticipatory anxiety and stress when in the presence of those triggers.^9^^,^^10^ A growing body of evidence suggests that living in a home affected by dampness and mold may be a contributor to poor mental health in the general population.^11^ Several epidemiological studies have reported associations between damp housing and depressive symptoms,^12^^,^^13^ psychological distress,^14^ and overall mental wellbeing.^15^^-^^17^ Given dampness and mold as well as poor mental health, especially anxiety are both respiratory triggers,^18^^,^^19^ it is plausible that a stronger mental health effect may be observed in this subgroup. However, no extant studies have examined the extent to which this association is modified by the presence of a respiratory condition such as asthma, and whether there is a higher risk of poor mental health upon exposure for people with a respiratory condition.

To address this gap, we used a large population-based sample to evaluate whether the association between damp housing and poor mental health is worse for people living with a respiratory condition. Understanding these relationships may not only provide a more comprehensive understanding of the damp housing-mental health nexus but also help to identify vulnerable populations and target interventions and prevention strategies.

## Methods

### Data source and population

Data from the British Household Panel Survey (BHPS) were used for this analysis. The BHPS is a nationally representative longitudinal study of 10 000 individuals aged 15 years or older from 5000 households in the United Kingdom, collecting data from 1991 to 2009.^20^ A total of 1500 additional households were added from Scotland and Wales in 1999, and 2000 households from Northern Ireland were added in 2001.^20^ Data were collected using interviews and self-completion questionnaires. The analytical sample for the present study consists of respondents aged 15 years and older who were followed for at least 2 consecutive waves between waves 6 and 18.

### Mental health outcome

Symptoms of mental health were derived from the General Health Questionnaire (GHQ). The GHQ is a commonly-used screening tool for general mental health, evaluating symptoms of psychological distress, encompassing anxiety, depression, somatic symptoms, and social dysfunction.^21^ General Health Questionnaire scores range from 0 (no/few symptoms of psychological distress) to 12 (highly symptomatic of psychological distress).^21^ The present study used a binary form of the GHQ, with a score below 3 coded as good mental health, and a score of 3 or more coded as poor mental health, based on previous evidence that a score of 3 is a useful cutoff to assess mental health in nonclinical populations.^22^^-^^25^

### Damp housing exposure

Exposure to damp housing was measured using a binary variable, defined as whether the participant responded “yes” to having any of the following indicators of building dampness: condensation, leaky roof, damp walls and/or floors, and rot in windows and/or floors. Each of these indicators was measured every wave from wave 6. While self-reported, our composite measure combining various specific indicators of dampness and mold reduces the risk of measurement error. Validation studies suggest that there is a close to 70% likelihood of correctly classifying cases of dampness and mold exposure using self-report measures.^26^ In particular, combining multiple measures of dampness exposure further reduces the likelihood of measurement error, and increases diagnostic power and discriminatory accuracy, particularly in identifying severe mold cases.^26^

### Respiratory conditions

Individuals were classified as having a respiratory condition if they reported: “chest/breathing problems, asthma, bronchitis” when asked the following question: “Do you have any of the health problems or disabilities listed on this card?”

### Covariates

Models were controlled for a range of covariates. Individual demographic covariates included age (years), sex (male/female) country of residence (England/Wales/Scotland/Northern Ireland), highest qualification (degree/other higher degree/A-level or equivalent/GCSE or equivalent/other qualification/no qualification), and employment status (paid employment/unemployed/self-employed/retired/student, apprentice, or trainee/maternity leave/family care or home/long-term sick or disabled/other). We additionally controlled for new diagnosis of presence of any long-term health condition other than respiratory (eg, diabetes, epilepsy, migraine, heart/blood pressure problems), using a binary yes/no variable. Household-related covariates included annual household income (£), household type (single nonelderly/single elderly/couple with no children/couple with dependent children/couple with nondependent children/lone parent with dependent children/lone parent with nondependent children/2 or more unrelated adults/other), residential tenure (owned outright/owned or being bought with mortgage/shared ownership (part owned, part rented)/rented/rent-free), adequacy of home heating (adequate/inadequate), and persons per room (a continuous variable assessing the presence of crowding, calculated as the number of persons in a household divided by number of rooms in the home excluding kitchens, bathrooms, and any rooms that are let or sublet).^27^

### Statistical analysis

All statistical analyses were performed using Stata SE 18.0 (StataCorp., College Station, TX, USA). To describe the analytic sample, the characteristics of the sample were calculated, recording the mean and SD of continuous variables and the number of person-years and percentage for categorical variables. We then used fixed effects logistic regression models to estimate the average within-person change in the relative odds of poor mental health, stratified by respiratory condition. Fixed effects models estimate the change in the outcome within people as their exposure status changes rather than between people, controlling for time-invariant individual-level factors.^28^ The models therefore address bias from both measured and unmeasured time-invariant confounding as between-person effects are discarded and the individual acts as their own control.^29^ This method effectively removes all between-individual variation, focusing solely on within-individual changes over time, allowing for causal inference under weaker assumptions than standard regression approaches.^30^ For example, time-invariant characteristics such as ethnicity are fully controlled for as this does not change within a person over time. Fixed effects models have been instrumental in assessing the effects of housing conditions, such as energy poverty,^31^ housing affordability,^32^ climate disasters,^33^ and environmental noise exposure,^34^ on health, reducing confounding and thereby allowing for causal inference. The basic fixed effects logistic regression model was specified as follows, noting that the outcome is dichotomous:


$$ PR\left({y}_{it}\!=\!1\right)\!=\!\frac{\exp \left({\alpha}_i+\beta{\mathrm{Damp}}_{it}+{\beta}_1{x}_{1 it}+{\beta}_2{x}_{2 it}\dots +{\beta}_k{x}_{kit}+{\varepsilon}_{it}\right)}{1+\exp \left({\alpha}_i+\beta{\mathrm{Damp}}_{it}+{\beta}_1{x}_{1 it}+{\beta}_2{x}_{2 it}\dots +{\beta}_k{x}_{kit}+{\varepsilon}_{it}\right)}, $$

where *y_it_* represents individual *i*’s psychological distress classification at time *t*, ${\alpha}_i$ is the unobserved individual-level component, Damp*_it_* is individual i’s exposure to damp housing at time *t*, *k* represents the included covariates, and $\varepsilon$*_it_* represents an error term that varies across individuals over time. To ensure valid statistical inference, we used robust standard errors clustered at the individual level, and time-varying covariates were included in the models to account for potential time-dependent confounding.

The models were adjusted for all covariates. The models were stratified by respiratory health at the time of baseline exposure measurement (ie, wave 6 for those who entered the survey prior to the first instance of damp housing measurement, or the first wave that someone participated in). Next, effect modification of the association between damp housing exposure and mental health was examined by 2 models including a statistical interaction between exposure to damp and mold and having a respiratory condition in a fixed effects logistic regression model, adjusted for covariates. The first model’s interaction term tested for effect modification by a change in respiratory condition, for example, receiving a diagnosis of a respiratory condition when previously not diagnosed. The second model’s interaction term tested for effect modification by diagnosis of a respiratory condition at baseline. Effect measure modification was assessed by examining the interaction fixed effects.

A series of sensitivity analyses were conducted to check the robustness of our effect estimates. First, to see if any observed effect modification was due to poor health in general, rather than poor respiratory health, we ran stratified analyses with presence of a chronic condition as the effect modifier. We also ran the stratified analyses using the continuous form of the GHQ, to see if effect modification was observed irrespective of the outcome cutoff point. To assess the possibility and impact of nondifferential misclassification of the effect modifier (eg, people not reporting a CRC in a wave after they had already reported having been diagnosed with one), we assessed the transition probabilities of the variable. We then re-conducted the stratified analyses with the sample restricted to people whose respiratory condition status did not change across their participation in the panel.

### Missing data

Missing data were present for the exposure, outcome, interaction variable and, to a lesser extent, the covariates (Table S1). The distribution of covariates was compared between participants with and without missing observations to determine whether incomplete data was associated with the values of missing variables. Participants with incomplete data reported lower education, lower rates of residency in England, and lower rates of paid employment (Table S2), suggesting that the data were not missing completely at random. Table S3 details the patterns of entry and exit in and out of the panel across wave 6-18. Across each wave, the percentage of individuals included at a wave that were also present in the next wave constituted approximately 90%. The biggest number of entries into the panel took place at wave 9 (35% first observation) and wave 11 (22% first observation). Total loss to follow-up constituted approximately 6%-8% of observations that exited and were not followed up again for each wave, apart from wave 11, which saw a loss to follow-up rate of 15%. Another 1%-3% per wave were not observed in the next but reappeared in future waves. The group of participants lost to follow-up by wave 18 had slightly higher rates of exposure to damp housing and of having symptoms of psychological distress (Table S4). This group also had lower average annual household income, higher rates of single elderly households and households comprised of 2 or more unrelated adults, higher rates of people with no qualifications, lower rates of outright home ownership and higher rates of shared ownership, and lower rates of paid employment (Table S4). Multiple imputation (MI) using chained equations with 50 imputations was performed to optimize the validity of the findings. The imputation models included all variables in the target analysis. The imputed dataset’s analysis was compared with the nonimputed dataset and the subset of participants with data for all waves to assess the impact of missing data and attrition.

## Results

### Descriptive statistics

The analytic sample comprised 186 389 observations. At baseline (wave 6), 9189 observations were included, increasing to 14 188 at wave 18. 25 066 (13.4%) observations came from people with respiratory conditions, and the remaining 161 323 came from people without respiratory conditions (Table 1). Across all person-years, people with respiratory conditions were, on average, 4 years older than those without respiratory conditions (49 vs 45 years), and had approximately less average annual income (£20 483 vs £23 873) compared with their counterparts without a respiratory condition. A greater proportion of people with respiratory conditions reported symptoms of psychological distress (38% vs 25%) and damp housing exposure (24% vs 20%). Both groups were broadly comparable in terms of sex, household type, country of origin, and adequacy of home heating. The group with a respiratory condition had a higher proportion of people with no qualifications (33% vs 21%), and a lower proportion of people in paid employment (37% vs 52%). The respiratory condition group also had a higher proportion of people in retirement (31% vs 19%) and on long-term disability (11% vs 4%). People living with a respiratory condition had a lower proportion of outright homeowners (62% vs 75%), with a higher proportion of people living in a home with shared ownership (36% vs 23%). Finally, people living with a respiratory condition were in poorer health than their counterparts, with higher prevalence of chronic conditions other than respiratory observed (75% vs 53%).

**Table 1:** Comparison of groups with/without respiratory conditions.

	Respiratory condition (person-years = 25 066)		No respiratory condition (person-years = 161 323)	
Variable	**Mean**	**SD**	**Mean**	**SD**
Age (years)	49.43	19.99	45.01	18.36
Household annual net income (£)	20483.21	14605.18	23 873	16405.15
Crowding (persons per room)	0.63	0.32	0.64	0.32
Variable	**Person-years**	**%**	**Person-years**	**%**
GHQ Score				
Below 3	15 564	62.09	121 612	75.38
3 or more	9502	37.91	39 711	24.62
Damp housing exposure				
No	19 120	76.30	129 671	80.38
Yes	5946	23.70	31 652	19.62
Sex				
Male	10 835	43.23	73 695	45.68
Female	14 231	56.77	87 628	54.32
Household type				
Single nonelderly	1885	7.52	11 208	6.95
Single elderly	3149	12.56	11 633	7.21
Couple no children	7796	31.10	47 130	29.21
Couple: dep children	6001	23.94	49 977	30.98
Couple: non-dep children	2779	11.09	20 859	12.93
Lone par: dep children	1301	5.19	8070	5.00
Lone par: non-dep children	1110	4.43	6279	3.89
2+ unrelated adults	501	2.00	3368	2.09
Other households	544	2.17	2799	1.74
Highest qualification				
Degree	2263	9.03	21 262	13.18
Other higher degree	1854	7.40	14 181	8.79
A-level or equivalent	4918	19.62	34 504	21.39
GCSE or equivalent	4988	19.90	40 462	25.08
Other qualification	2722	10.86	16 431	10.19
No qualification	8321	33.20	34 483	21.38
Tenure				
Owned outright	15 589	62.19	120 301	74.52
Owned/being bought on mortgage	78	0.31	647	0.40
Shared ownership (part owned/rented)	8969	35.78	37 650	23.34
Rented	379	1.51	2289	1.42
Rent free	51	0.20	436	0.27
Country				
England	13 888	55.41	89 931	55.75
Wales	4424	17.65	24 841	15.40
Scotland	4327	17.26	28 010	17.36
Northern Ireland	2427	9.68	18 541	11.49
Long-term health condition other than respiratory				
No	6260	24.97	76 174	47.22
Yes	18 806	75.03	85 149	52.78
Home heating				
Adequate	23 620	94.23	154 493	95.77
Inadequate	1446	5.77	6830	4.23
Employment status				
Paid employment	9214	36.76	84 501	52.38
Unemployed	944	3.77	5428	3.36
Self-employed	1127	4.50	11 370	7.05
Retired	7693	30.69	31 299	19.40
Student/apprentice/trainee	1376	5.49	10 247	6.35
Maternity leave	83	0.33	724	0.45
Family care/home	1877	7.49	11 332	7.02
LT sick or disabled	2638	10.52	5665	3.51
Other	114	0.45	757	0.47

### Results of regression analyses

**Figure 1:**
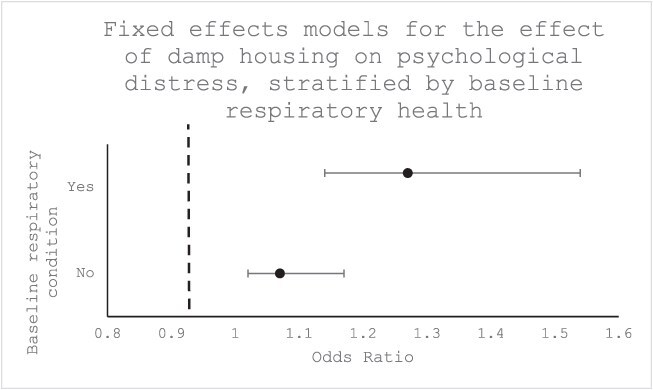
Fixed effects models for the effect of damp housing on psychological distress, stratified by baseline respiratory health.

In stratified fixed effects regression analyses (comparing the scenario of people with and without respiratory health conditions becoming exposed to damp housing, Figure 1), exposure to damp housing was associated with a 1.27-fold increase in the relative odds of poor mental health (95% CI: [1.14, 1.41], *P* < .01) for people living with a chronic respiratory health condition; much higher than the 1.07-fold estimated for people who did not report a respiratory condition (95% CI: [1.02, 1.12], *P* = .01, Table 2).

**Table 2:** Results of fixed effects logistic regression model analyzing the effect of damp housing on mental health stratified by diagnosis with a respiratory condition at baseline, fully adjusted.

**Respiratory condition at baseline**	**Odds ratio**	**95% CI**	***P*-value**
No	1.07	1.02, 1.12	0.01
Yes	1.27	1.14, 1.41	<0.01

**Figure 2:**
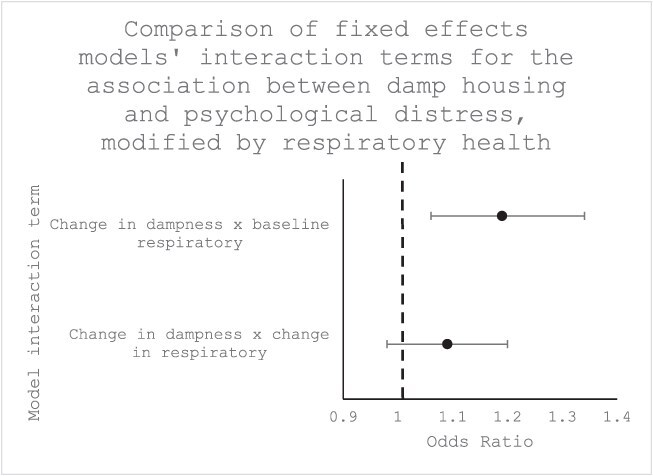
Comparison of interaction terms from fixed effects logistic regression models analyzing the effect of damp housing on mental health, modified by baseline respiratory health.

In the fixed effects analysis accounting for the damp/respiratory condition interaction (Table 3, Figure 2) (representing the scenario of people newly exposed to mold and damp and also newly diagnosed with a respiratory condition), the overall odds ratio for the association between damp housing and mental health was 1.08 (95% CI: [1.03, 1.13], *P* < .01). The odds ratio for the interaction term was 1.09 (95% CI: [0.98, 1.20], *P* = .10), showing weak evidence for effect modification by presence of a respiratory condition when the values of both the exposure and effect measure modifier changed. However, in the second model (representing the scenario of people newly exposed to mold and damp who had a diagnosis of a respiratory condition at baseline), the odds ratio for the interaction term was 1.19 (95% CI: [1.06, 1.34], *P* < .01), showing strong evidence for effect modification by presence of a respiratory condition when the value of the effect modifier was fixed at its baseline level.

**Table 3:** Comparison of interaction terms from 2 fixed effects logistic regression models analyzing the effect of damp housing on mental health with damp/respiratory and damp/baseline respiratory interaction terms, fully adjusted.

**Model**	**Interaction term odds ratio**	**95% CI**	***P*-value**
Change in damp × Change in respiratory condition	1.09	0.98, 1.20	.1
Change in damp × Baseline respiratory	1.19	1.06, 1.34	<.01

### Sensitivity analysis

**Figure 3:**
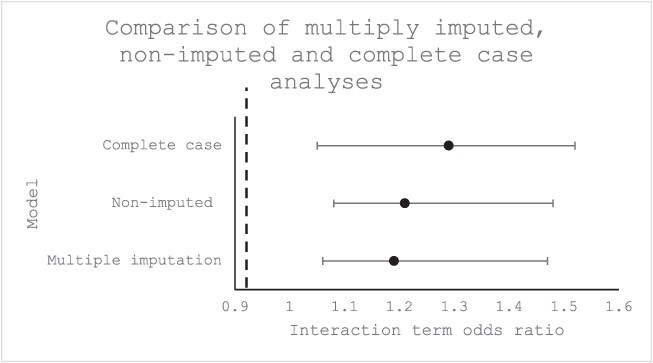
Comparison of interaction terms in multiply imputed, nonimputed, and complete case fixed effects logistic regression models for the association between damp housing and psychological distress, modified by baseline respiratory health.

When we tested to see how the effects compared in people with and without chronic conditions at baseline (Tables 4 and 5), there were negligible differences in mental health effects. This indicates that the effect modification observed is for people with respiratory conditions specifically and not for people with any chronic health condition. Effect modification was also observed when using the continuous form of the outcome (Tables 6 and 7). Between waves, there was good cross-wave agreement for those who reported a CRC—there was a high probability of reporting the same condition in subsequent waves (Table 8). Restriction of the sample to those whose respiratory condition status did not change across their participation in the sample generated similar results to the main analyses (Table 9). In the nonimputed dataset (Figure 3, Tables S5 and S6), there was a lower level of certainty reported for people without respiratory conditions, and effect estimates were greater for people with respiratory conditions compared with the results from the multiply imputed dataset. Similar results were found for the subset of participants who had data for all 13 waves (Tables S7 and S8), with effect estimates significantly higher in the complete case data subset compared with the imputed dataset.

**Table 4:** Sensitivity analysis—fixed effects logistic regression models for the association between damp housing and mental health, stratified by presence of a chronic condition at baseline.

	**OR**	**95% CI**	***P*-value**
No chronic condition	1.06	1.00, 1.13	.07
Chronic condition	1.14	1.07, 1.20	<.01

**Table 5:** Sensitivity analysis—comparison of fixed effects logistic regression models for the association between damp housing and mental health, with damp/chronic condition interaction terms.

**Independent variable**	**Odds ratio**	**95% CI**	***P*-value**
Change in damp/change in chronic condition	1.02	0.94, 1.10	.66
Change in damp/baseline chronic condition	1.08	1.00, 1.18	.04

**Table 6:** Sensitivity analysis—fixed effects linear regression models for the association between damp housing and mental health, stratified by presence of a respiratory condition.

	**Coeff.**	**95% CI**	***P*-value**
No respiratory condition	0.08	0.04, 0.12	<0.01
Respiratory condition	0.22	0.11, 0.33	<0.01

**Table 7:** Sensitivity analysis—comparison of interaction terms from fixed effects linear regression models for the association between damp housing and mental health, with change in damp/change in respiratory and change in damp/baseline respiratory condition interaction terms.

**Independent variable**	**Interaction term coefficient.**	**95% CI**	***P*-value**
Change in damp × Change in respiratory	0.11	0.02, 0.20	.02
Change in damp × Baseline respiratory	0.13	0.04, 0.23	.01

**Table 8:** Sensitivity analysis—transition probabilities for diagnosis of a chronic respiratory condition.

	**No respiratory condition (T + 1)**	**Respiratory condition (T + 1)**
No respiratory condition (T)	95.73	4.27
Respiratory condition (T)	26.27	73.73

T = current wave; T + 1 = subsequent wave.

**Table 9:** Sensitivity analysis—fixed effects logistic regression models for the association between damp housing and mental health, stratified by presence of a respiratory condition at baseline, restricting the sample to those whose respiratory condition status did not change across their participation in the panel.

	**Odds ratio**	**95% CI**	***P*-value**
No respiratory condition	1.06	1.01, 1.12	0.02
Respiratory condition	1.19	1.00, 1.42	0.06

## Discussion

To our knowledge, this is the first study to investigate whether the mental health effects of residential dampness exposure differ between people with and without a respiratory condition such as asthma, COPD, or chronic bronchitis. We found that, while both groups had a statistically significant increase in the odds of poor mental health with exposure to damp, the association in people with a respiratory condition was of a greater magnitude. However, the results of the 2 fixed effects models with interaction terms suggest that it is the presence of a pre-existing respiratory condition, and not change respiratory health, that generates the finding.

There are several potential explanations for the difference in effect estimates between those with and without a respiratory condition at baseline. First, exposure to indoor dampness and mold is significantly associated with onset and exacerbation of respiratory symptoms in people with conditions such as asthma and COPD.^4^^,^^35^ Therefore, the increased symptom burden, and the stress of managing those symptoms, may be the reason for increased odds of poor mental health in individuals with respiratory conditions compared with those without such a condition.

It is also plausible that people with respiratory conditions may be more likely to suffer from stress and anxiety due to elevated awareness of indoor dampness and mold as triggers for attacks and exacerbations of their health condition. Perceived asthma triggers, including allergens, have been observed to play a role in anxiety and depression.^9^ This has been shown in relation to allergenic pollen, whereby awareness of pollen levels is associated with poorer mental health.^10^ Qualitative studies of asthmatic individuals find that awareness of environmental triggers, such as mold, lead to lifestyle adjustments and avoidance methods, fear and anxiety around potential triggers, and stress regarding the social, financial, and environmental barriers to preventing or managing a flare-up of a respiratory condition.^8^^,^^36^^,^^37^

Finally, it may be that people with respiratory conditions who are exposed to indoor dampness and mold are using reliever medications on a regular basis to mitigate the effects of the exposure. Frequent use of these medications is associated with poor mental health. For example, Salbutamol and other short-acting beta agonist (SABA) medications (used for quick relief) have nervous system effects and can induce anxiety symptoms.^38^ Similarly, systemic (oral, injected, or intravenous) corticosteroids, used to relieve asthma exacerbations that are not controlled by preventive inhalers and SABAs, have known psychiatric side effects.^39^ It is therefore probable that, by reducing exposure to indoor dampness, the subsequent reduction in asthma medication usage for those for whom damp is a trigger for their symptoms may mitigate the mental health effects of damp housing in people with respiratory conditions.

The results of our study suggest that interventions targeting damp housing for people with respiratory conditions may have a 2-fold benefit, on both physical and mental health. Evidence from randomized controlled trials find that respiratory symptoms improve and medication use declines when dampness and mold in the homes of asthmatic patients are addressed.^40^^-^^42^ Given the improvement in respiratory symptoms associated with allergen reduction, it is plausible that mental health could additionally improve. This emphasizes the need for a comprehensive, validated screening tool that clinicians can use to evaluate environmental exposures at home in patients with respiratory conditions, so they can provide advice to and advocate for patients exposed to poor indoor environments.^43^ Additionally, given that the relationship between CRCs and mental health is bidirectional,^19^^,^^44^^,^^45^ reducing the mental health burden of damp housing may reduce respiratory symptoms, and vice versa. However, no trials have included mental health as an outcome of addressing dampness and mold. Including mental health as an outcome in future trials of dampness and mold remediation may give us a more holistic understanding of the benefits of addressing asthma triggers in the home.

Our study has some limitations. Presence of a respiratory condition was self-reported and not confirmed by an independent healthcare professional, which may be subject to recall bias. Nonetheless, a comparison of self-report and physician diagnoses ascertained via medical records shows that self-reported obstructive respiratory conditions are highly correlated with physician diagnoses.^46^ Additionally, respiratory conditions were not separated out, so we were not able to ascertain whether the association differed depending on the specific respiratory condition. Future work should use objective outcomes such as doctor-diagnosed asthma and diagnostic tools (eg, spirometry, peak flow, bronchodilator responsiveness test) to confirm presence of a respiratory condition. Moreover, dampness exposure was also self-reported, limiting our ability to accurately quantify the level of exposure due to uncertainty around estimates. However, the use of a combined measure of dampness exposure decreased the likelihood of measurement error, given the higher diagnostic accuracy of combined measures of dampness and mold exposure.^26^ Future studies would benefit from using more objective measures of exposure, for example, through home inspection or through indoor microbiological analysis. Finally, due to the nature of an observational study, we cannot disregard the possibility of residual confounding due to unmeasured factors that may influence the association.

## Conclusion

This is the first study to provide evidence that people with respiratory conditions might be more vulnerable to poor mental health due to damp housing than those without. Given this finding, interventions to reduce dampness and mold in the homes of people with respiratory conditions may have a 2-fold benefit, improving both respiratory symptoms and mental health.

## Supplementary Material

Web_Material_kwag042

## Data Availability

Original data from the British Household Survey and the UK Household Longitudinal Study were collected by the University of Essex, Institute for Social and Economic Research. Data is available for download from the UK Data Service.
