# Supplementary material for: The effect of damp housing on psychological distress: does respiratory health matter?
Source: Am J Epidemiol. 2026 Feb 28;195(5):1292–9. doi: 10.1093/aje/kwag042 (PMC13149003; doi:10.1093/aje/kwag042)
Supplement: Web_Material_kwag042 [file web_material_kwag042.zip › AJE_SuppMaterial.docx]

**Supplementary Material**

**The effect of damp housing on psychological distress: does respiratory health matter?**

Maria Rosa Gatto, Ang Li, Erika Martino, and Rebecca Bentley

**Table S1.** Description of missing data (total person-years = 186,389) – Page 2

**Table S2.** Comparison of observations with and without missing data (total person-years = 186,389) – Page 3

**Table S3.** Patterns of entry and exit into and out of the panel – Page 4

**Table S4.** Comparison of individuals lost to follow-up and those not lost to follow-up at wave 18. – Page 5

**Table S5.** Sensitivity analysis – Fixed effects logistic regression models for the association between damp housing and mental health, stratified by presence of a respiratory condition, using non-imputed dataset. – Page 6

**Table S6.** Sensitivity analysis – Comparison of interaction terms from two fixed effects logistic regression models for the association between damp housing and mental health, with change in damp/change in respiratory and change in damp/baseline respiratory interaction terms, using non-imputed dataset. – Page 6

**Table S7.** Sensitivity analysis – Fixed effects logistic regression models for the association between damp housing and mental health, stratified by presence of a respiratory condition, for people with complete data across waves 6-18. – Page 6

**Table S8.** Fixed effects logistic regression models for the association between damp housing and mental health, with change in damp/change in respiratory and change in damp/baseline respiratory interaction terms, for people with complete data across waves 6-18. – Page 6

**Table S1: Description of missing data (total person-years = 186,389)**

| **Variable** | **Person-years missing** | **% Missing** |
| --- | --- | --- |
| Mental health | 11,897 | 6·38 |
| Damp housing exposure | 6,215 | 3·33 |
| Respiratory conditions | 2,810 | 1·51 |
| Highest qualification | 3,551 | 1·91 |
| Sex | 81 | 0·04 |
| Chronic health conditions | 2,810 | 1·51 |
| Age | 6 | <0·01 |
| Household type | 0 | 0 |
| Tenure | 3,654 | 1·96 |
| Household income | 5,225 | 2·80 |
| Country | 1,363 | 0·73 |
| Employment status | 40 | 0·02 |
| Crowding | 4,008 | 2.15 |

**Table S2: Comparison of observations with and without missing data (total person-years = 186,389)**

|  | **Complete observations (n = 150,138)** | **Observations with missing data (n = 36,170)** |
| --- | --- | --- |
| **Covariates (continuous)** | **Mean (SD)** | **Mean (SD)** |
| Age (years) | 45·44 (18·39) | 46·29 |
| Household annual net income (£) | 23,804·71 (16,492·43) | 21,570·21 (14,715·66) |
| Crowding (persons per room) |  |  |
| **Covariates (categorical)** | **Person-years (%)** | **Person-years (%)** |
| Sex |  |  |
| Male | 68,204 (45·43) | 16,276 (45) |
| Female | 81,934 (54·57) | 19,894 (55) |
| Household type |  |  |
| Single non-elderly | 10,087 (6·72) | 3,006 (8·29) |
| Single elderly | 11,331 (7·55) | 3,451 (9·52) |
| Couple no children | 45,010 (29·98) | 9,916 (27·35) |
| Couple: dep children | 45,688 (30·43) | 10,290 (28·39) |
| Couple: non-dep children | 19,034 (12·68) | 4,604 (12·70) |
| Lone par: dep children | 7,523 (5·01) | 1,848 (5·10) |
| Lone par: non-dep children | 5,771 (3·84) | 1,618 (4·46) |
| 2+ unrelated adults | 3,100 (2·06) | 769 (2·12) |
| Other households | 2,594 (1·73) | 749 (2·07) |
| Highest qualification |  |  |
| Degree | 19,381 (12·91) | 3,699 (11·31) |
| Other higher degree | 13,069 (8·70) | 2,616 (8·00) |
| A-level or equivalent | 32,457 (21·62) | 6,238 (19·08) |
| GCSE or equivalent | 36,983 (24·63) | 7,575 (23·17) |
| Other qualification | 15,479 (10·31) | 3,313 (10·13) |
| No qualification | 32,769 (21·83) | 9,259 (28·31) |
| Tenure |  |  |
| Owned outright | 110,309 (73·47) | 23,031 (70·65) |
| Owned/being bought on mortgage | 605 (0·40) | 105 (0·32) |
| Shared ownership (part owned/rented) | 36,812 (24·52) | 8,835 (27·10) |
| Rented | 2,036 (1·36) | 533 (1·64) |
| Rent free | 376 (0·25) | 93 (0·29) |
| Country |  |  |
| England | 86,840 (57·84) | 16,345 (46·85) |
| Wales | 23,065 (15·36) | 5,982 (17·15) |
| Scotland | 25,791 (17·18) | 6,238 (17·88) |
| Northern Ireland | 14,442 (9·62) | 6,323 (18·12) |
| Long-term illness/condition |  |  |
| No | 60,195 (40·09) | 13,387 (40·03) |
| Yes | 89,943 (59·91) | 20,054 (59·97) |
| Lack of adequate heating |  |  |
| No | 143,616 (95·66) | 28,513 (9·10) |
| Yes | 6,522 (4·34) | 1,470 (4·90) |
| Employment status |  |  |
| Paid employment | 76,766 (51·13) | 16,930 (46·75) |
| Unemployed | 4,968 (3·31) | 1,403 (3·87) |
| Self-employed | 10,133 (6·75) | 2,359 (6·51) |
| Retired | 30,699 (20·45) | 8,286 (22·88) |
| Student/apprentice/trainee | 9,212 (6·14) | 2,406 (6·64) |
| Maternity leave | 675 (0·45) | 132 (0·36) |
| Family care/home | 10,599 (7·06) | 2,608 (7·20) |
| LT sick or disabled | 6,406 (4·27) | 1,896 (5·24) |
| Other | 680 (0·45) | 191 (0·53) |

**Table S3: Patterns of entry and exit into and out of the panel**

| Wave^1^ | Total^2^ | Inc1^3^ | Entry^4^ | First^5^ | Re-enter^6^ | Inc2^7^ | Exit^8^ | Last^9^ | Re-exit^10^ |
| --- | --- | --- | --- | --- | --- | --- | --- | --- | --- |
| 6 | 9,189 | 0 | 9,189 | 9,189 | 0 | 8,498 | 691 | 558 | 133 |
| 7 | 10,870 | 8,498 | 2,372 | 2,372 | 0 | 9,945 | 925 | 734 | 191 |
| 8 | 10,624 | 9,945 | 679 | 595 | 84 | 9,780 | 844 | 621 | 223 |
| 9 | 15,275 | 9,780 | 5,495 | 5,356 | 139 | 13,770 | 1,505 | 1,121 | 384 |
| 10 | 15,307 | 13,770 | 1,537 | 1,336 | 201 | 13,969 | 1,338 | 1,020 | 318 |
| 11 | 18,392 | 13,969 | 4,423 | 4,081 | 342 | 15,082 | 3,310 | 2,886 | 424 |
| 12 | 16,223 | 15,082 | 1,141 | 842 | 299 | 14,615 | 1,608 | 1,215 | 393 |
| 13 | 15,855 | 14,615 | 1,240 | 842 | 398 | 14,323 | 1,532 | 1,202 | 330 |
| 14 | 15,459 | 14,323 | 1,136 | 707 | 429 | 14,181 | 1,278 | 1,027 | 251 |
| 15 | 15,307 | 14,181 | 1,126 | 765 | 361 | 14,148 | 1,159 | 995 | 164 |
| 16 | 15,092 | 14,148 | 944 | 642 | 302 | 13,788 | 1,304 | 1,095 | 209 |
| 17 | 14,608 | 13,788 | 820 | 627 | 193 | 13,420 | 1,188 | 1,188 | 0 |
| 18 | 14,188 | 13,420 | 768 | 496 | 272 | 0 | 14,188 | 14,188 | 0 |

^1^Wave – wave of the panel

^2^Total – total number of individuals included in the specified wave

^3^Inc1 – Number of individuals at the specified wave that were also present at the previous wave

^4^Entry – Number of individuals at the specified wave that were not present at wave 6

^5^First – Number of individuals at the specified wave who show up for the first time at that wave

^6^Re-enter – Number of individuals at the specified wave that are re-entering at that wave

^7^Inc2 – Number of individuals at the specified wave that are also present in the next wave

^8^Exit – Number of individuals at the specified wave that are not present in the next wave

^9^Last – Number of individuals at the specified wave that are not present at any future time

^10^Re-exit – Number of individuals at the specified wave not present in the next wave that appear in later waves

**Table S4: Comparison of individuals lost to follow-up and those not lost to follow-up at wave 18**

|  | **Lost to follow-up- by wave 18 (person-years = 52,401, observations = 13,662)** | **Not lost to follow-up at wave 18 (person-years = 133,982, observations = 14,188)** |
| --- | --- | --- |
| **Continuous variables** | **Mean (SD)** | **Mean (SD)** |
| Age (years) | 46.15 (21.47) | 45.39 (17.42) |
| Household annual net income (£) | 20,369.05 (15,494.52) | 24,595.11 (16,344.53) |
| Crowding (persons per room) | 0.67 (0.34) | 0.63 (0.31) |
| **Categorical variables** | **Person-years (%)** | **Person-years (%)** |
| Dampness |  |  |
| No | 38,010 (76.22) | 105,786 (81.18) |
| Yes | 11,857 (23.78) | 24,521 (18.82) |
| GHQ Score |  |  |
| Under 3 | 34,360 (65.57) | 94,874 (70.81) |
| 3 or more | 18,041 (34.43) | 39,114 (29.19) |
| Respiratory condition |  |  |
| No | 43,448 (83.97) | 115,263 (87.43) |
| Yes | 8,295 (16.03) | 16,573 (12.57) |
| Sex |  |  |
| Male | 25,147 (48.00) | 59,333 (44.30) |
| Female | 27,239 (52.00) | 74,589 (55.70) |
| Household type |  |  |
| Single non-elderly | 3,474 (6.63) | 9,619 (7.18) |
| Single elderly | 6,054 (11.55) | 8,728 (6.51) |
| Couple no children | 14,296 (27.28) | 40,630 (30.32) |
| Couple: dep children | 13,436 (25.64) | 42,542 (31.75) |
| Couple: non-dep children | 6,558 (12.52) | 17,080 (12.75) |
| Lone par: dep children | 2,808 (5.36) | 6,563 (4.90) |
| Lone par: non-dep children | 2,292 (4.37) | 5,097 (3.80) |
| 2+ unrelated adults | 2,333 (4.45) | 1,536 (1.15) |
| Other households | 1,150 (2.19) | 2,193 (1.64) |
| Highest qualification |  |  |
| Degree | 4,909 (9.62) | 18,171 (13.78) |
| Other higher degree | 3,676 (7.21) | 12,009 (9.11) |
| A-level or equivalent | 10,251 (20.10) | 28,444 (21.58) |
| GCSE or equivalent | 11,806 (23.15) | 32,752 (24.84) |
| Other qualification | 5,044 (9.89) | 13,748 (10.43) |
| No qualification | 15,321 (30.04) | 26,707 (20.26) |
| Tenure |  |  |
| Owned outright | 31,149 (61.83) | 102,191 (77.21) |
| Owned/being bought on mortgage | 188 (0.37) | 522 (0.39) |
| Shared ownership (part owned/rented) | 17,982 (35.69) | 27,665 (20.90) |
| Rented | 911 (1.81) | 1,658 (1.25) |
| Rent free | 147 (0.29) | 322 (0.24) |
| Country |  |  |
| England | 28,968 (56.42) | 74,217 (55.52) |
| Wales | 7,161 (13.95) | 21,886 (16.37) |
| Scotland | 9,657 (18.81) | 22,372 (16.73) |
| Northern Ireland | 5,556 (10.82) | 15,209 (11.38) |
| Long-term illness/condition |  |  |
| No | 20,052 (38.75) | 53,530 (40.60) |
| Yes | 31,691 (61.25) | 78,306 (59.40) |
| Lack of adequate heating |  |  |
| No | 46,860 (94.01) | 125,269 (96.16) |
| Yes | 2,988 (5.99) | 5,004 (3.84) |
| Employment status |  |  |
| Paid employment | 22,398 (42.76) | 71,298 (53.22) |
| Unemployed | 2,486 (4.75) | 3,885 (2.90) |
| Self-employed | 3,091 (5.90) | 9,401 (7.02) |
| Retired | 13,337 (25.46) | 25,648 (19.15) |
| Student/apprentice/trainee | 4,448 (8.49) | 7,170 (5.35) |
| Maternity leave | 125 (0.24) | 682 (0.51) |
| Family care/home | 3,459 (6.60) | 9,748 (7.28) |
| LT sick or disabled | 2,762 (5.27) | 5,540 (4.14) |
| Other | 276 (0.54) | 595 (0.44) |

**Table S5: Sensitivity analysis – Fixed effects logistic regression models for the association between damp housing and mental health, stratified by presence of a respiratory condition, using non-imputed dataset**

|  | Odds Ratio | 95% CI | p-value |
| --- | --- | --- | --- |
| No respiratory condition | 1.03 | 0.99, 1.08 | 0.11 |
| Respiratory condition | 1.26 | 1.14, 1.41 | <0.01 |

**Table S6: Sensitivity analysis – Comparison of interaction terms from two fixed effects logistic regression models for the association between damp housing and mental health, with change in damp/change in respiratory and change in damp/baseline respiratory interaction terms, using non-imputed dataset**

| Independent Variable | Interaction Term Odds Ratio | 95% CI | p-value | Wald test p-value |
| --- | --- | --- | --- | --- |
| Damp x respiratory interaction term | 1.12 | 1.01, 1.23 | 0.03 | 0.03 |
| Damp x baseline respiratory interaction term | 1.21 | 1.08, 1.35 | 0.001 | <0.01 |

**Table S7: Sensitivity analysis – Fixed effects logistic regression models for the association between damp housing and mental health, stratified by presence of a respiratory condition, for people with complete data across waves 6-18**

|  | Odds Ratio | 95% CI | p-value |
| --- | --- | --- | --- |
| No respiratory condition | 1.06 | 0.99, 1.14 | 0.11 |
| Respiratory condition | 1.43 | 1.18, 1.73 | <0.01 |

**Table S8: Sensitivity analysis – Fixed effects logistic regression models for the association between damp housing and mental health, with change in damp/change in respiratory and change in damp/baseline respiratory interaction terms, for people with complete data across waves 6-18**

| Independent Variable | Interaction Term Odds Ratio | 95% CI | p-value | Wald test p-value |
| --- | --- | --- | --- | --- |
| Change in damp x change in respiratory | 1.04 | 0.88, 1.24 | 0.62 | 0.62 |
| Change in damp x baseline respiratory | 1.29 | 1.05, 1.28 | 0.01 | 0.01 |
